# Hydrogel Drug Delivery Systems and Liposomal Bupivacaine: Innovations and Future Perspectives in Pain Management

**DOI:** 10.3390/jcm14217824

**Published:** 2025-11-04

**Authors:** Kyung Kwan Lee, Wonwoo Jeong, Minsuk Chae

**Affiliations:** 1 Department of Bio-Chemical Engineering, Chosun University, 309 Pilmun-daero, Dong-gu, Gwangju 61452, Republic of Korea; kylee@chosun.ac.kr; 2 Wake Forest Institute for Regenerative Medicine, Wake Forest School of Medicine, Winston-Salem, NC 27157, USA; wonwoo.jeong@advocatehealth.org; 3 Department of Anesthesiology and Pain Medicine, College of Medicine, The Catholic University of Korea, Seoul 06591, Republic of Korea

**Keywords:** hydrogels, liposomal bupivacaine, pain management, sustained release, stimuli-responsive drug delivery

## Abstract

Pain management remains a major clinical challenge, as conventional opioids and local anesthetics suffer from short analgesic duration, systemic toxicity, and dependence risks. Advanced drug delivery systems, particularly hydrogels and liposomal bupivacaine, have emerged as promising solutions to address these limitations. Hydrogels, composed of cross-linked hydrophilic polymer networks, enable biocompatible, biodegradable, and sustained drug release, while liposomal bupivacaine encapsulates the anesthetic within lipid vesicles to prolong local analgesia and minimize systemic toxicity. The combination of these systems offers synergistic benefits, including extended drug retention, enhanced efficacy, and reduced opioid reliance. However, clinical translation remains constrained by formulation instability, high production costs, variable patient responses, and stringent regulatory requirements. This review provides a comprehensive overview of current advances in hydrogel and liposomal bupivacaine technologies, highlighting their clinical potential, ongoing challenges, and future directions toward safer, more effective, and personalized pain management strategies.

## 1. Introduction

Pain management continues to pose significant clinical challenges despite ongoing advancements in medical technologies and pharmacological therapies. Effective pain control is essential for enhancing patient quality of life, facilitating quicker recovery, and reducing the overall burden on healthcare systems [1]. Nevertheless, current analgesic approaches, particularly opioids and conventional local anesthetics, present considerable drawbacks. Opioids, while effective for severe pain, are associated with significant side effects, including respiratory depression, sedation, gastrointestinal disturbances, tolerance development, and a high risk of addiction and misuse [2]. Additionally, prolonged opioid use contributes to the ongoing global opioid crisis, posing substantial public health concerns. Inadequate pain management can lead to chronic pain syndromes, delayed postoperative recovery, increased hospital readmission rates, higher healthcare costs, and diminished patient satisfaction and quality of life [3,4]. These persistent challenges underscore the urgent necessity for innovative analgesic delivery systems that offer safe, effective, and sustained pain relief while minimizing adverse effects.

Conventional local anesthetics, though widely used in clinical practice for temporary pain relief, exhibit several significant limitations that restrict their optimal utility. A primary limitation is their relatively short duration of analgesic effect, typically lasting only a few hours, requiring frequent re-administration or continuous infusion via invasive catheters [5]. These repeated interventions not only exacerbate patient discomfort but also increase procedural risks, such as infection, nerve damage, bleeding complications, and catheter-related complications. Furthermore, traditional local anesthetics administered at higher or repeated doses carry substantial risks of systemic toxicity, manifesting as neurological symptoms (e.g., seizures, dizziness, and confusion) and potentially severe cardiovascular complications, including arrhythmias, hypotension, and cardiac arrest. Additionally, the rapid systemic absorption and redistribution away from the target tissue often result in inconsistent and unpredictable analgesic outcomes, reducing overall clinical reliability. These cumulative drawbacks emphasize the critical need for innovative, sustained-release local anesthetic formulations specifically designed to overcome existing pharmacological and clinical limitations [6].

In response to these challenges, novel drug delivery systems, particularly hydrogels, and advanced local anesthetic formulations, notably liposomal bupivacaine, have emerged as promising and transformative approaches within pain management. Hydrogels, composed of cross-linked hydrophilic polymer networks, have attracted significant research attention due to their unique characteristics, such as high biocompatibility, biodegradability, sustained and controllable drug release, and responsiveness to physiological stimuli such as pH, temperature, and enzymatic activity. These distinctive features enable prolonged, localized drug delivery directly at the target site, thereby reducing systemic exposure, minimizing toxicity, and significantly decreasing the frequency of drug administration [7,8]. Concurrently, liposomal bupivacaine encapsulating bupivacaine within lipid-based vesicles offers a significantly extended analgesic duration and an improved pharmacokinetic profile. This encapsulation prolongs local anesthetic effects, stabilizes plasma concentrations, and considerably reduces the incidence of systemic adverse reactions. The combined rationale behind utilizing hydrogels and liposomal formulations in clinical practice arises from their synergistic potential to enhance analgesic efficacy, improve patient comfort and safety, and effectively address critical unmet needs within modern pain management paradigms [9,10].

The objective of this narrative review is to provide a comprehensive and current overview of innovations and advancements in hydrogel-based drug delivery systems and the novel local anesthetic formulation, liposomal bupivacaine, within the context of pain management. Specifically, this review evaluates the rationale behind adopting these technologies, explores their pharmacological profiles, assesses clinical evidence supporting their application, and discusses the implications for integration into clinical practice. The article begins with an overview of existing challenges in pain management and the limitations of conventional analgesics, followed by a detailed examination of hydrogels, including their classification, properties, and clinical applications. Next, the pharmacological advantages, clinical efficacy, and safety of liposomal bupivacaine are discussed. The subsequent section explores the potential combined application of hydrogels and liposomal bupivacaine, highlighting their synergistic effects and reviewing experimental studies. Finally, technological innovations, current challenges, and future research directions are presented, offering valuable insights into the future landscape of effective and safe pain management strategies.

### Methods

This narrative review was conducted through a comprehensive literature search of peer-reviewed journals and online databases, including PubMed, Scopus, and Web of Science. The search covered articles published between 2008 and 2025 using keywords such as “hydrogel drug delivery”, “liposomal bupivacaine”, “local anesthetic”, “pain management”, and “sustained release systems”. Only English-language articles were included. Additional references were identified from the bibliographies of relevant reviews and experimental studies. The focus was placed on studies discussing formulation strategies, preclinical efficacy, clinical trials, and translational challenges of hydrogel-based and liposomal analgesic systems.

## 2. Overview of Hydrogels for Drug Delivery

### 2.1. Definition and Classification of Hydrogels

Hydrogels are three-dimensional, cross-linked networks of hydrophilic polymers capable of absorbing significant amounts of water or biological fluids while maintaining their structural integrity. Due to their high-water content and structural resemblance to natural tissues, hydrogels exhibit excellent biocompatibility, making them highly advantageous for biomedical and pharmaceutical applications, particularly in drug delivery systems. Hydrogels are broadly classified based on two main criteria: the nature of their crosslinking mechanisms (chemical vs. physical) and the origin of their constituent polymers (natural vs. synthetic) [11].

#### 2.1.1. Chemical vs. Physical Hydrogels

Chemical hydrogels are formed by covalent bonds between polymer chains, resulting in stable, rigid, and permanent network structures. The covalent crosslinking provides these hydrogels with mechanical robustness, controlled swelling properties, and resistance to degradation or dissolution under physiological conditions. Due to their structural stability, chemical hydrogels are ideal candidates for sustained-release drug delivery systems, ensuring prolonged drug release over extended periods, thus minimizing administration frequency and enhancing patient compliance. Examples of chemical crosslinking methods include radical polymerization, condensation reactions, and click chemistry, which offer precise control over hydrogel characteristics tailored to specific clinical needs [12,13,14].

In contrast, physical hydrogels are formed through reversible, non-covalent interactions such as hydrogen bonding, hydrophobic interactions, ionic bonds, or molecular entanglements. These interactions yield dynamic and reversible network structures, allowing physical hydrogels to respond to environmental stimuli, including changes in temperature, pH, ionic strength, and biochemical signals. Such responsiveness enables the development of intelligent, stimuli-sensitive drug delivery systems that can release therapeutic agents in a controlled and targeted manner. Thermosensitive hydrogels, such as those based on poly (N-isopropylacrylamide) (PNIPAAm), exhibit temperature-responsive sol–gel transitions that facilitate minimally invasive administration, while pH-sensitive hydrogels, such as those incorporating poly (acrylic acid) (PAA), enable drug release in response to physiological pH variations found in targeted tissues or pathological environments [15,16,17].

#### 2.1.2. Natural vs. Synthetic Hydrogels

Natural hydrogels are derived from biological polymers such as alginate, gelatin, collagen, chitosan, hyaluronic acid, and fibrin. These naturally occurring polymers inherently exhibit biocompatibility, biodegradability, low toxicity, and bioactivity, making them attractive for tissue engineering, regenerative medicine, and drug delivery applications. For instance, alginate hydrogels, derived from brown algae, demonstrate exceptional biocompatibility and gentle gelling conditions, making them suitable for encapsulating sensitive therapeutic agents and biological molecules. Collagen and gelatin-based hydrogels support cellular proliferation and tissue regeneration, thereby promoting healing and enhancing therapeutic outcomes in drug delivery applications [18,19,20].

Synthetic hydrogels, on the other hand, are constructed from synthetic polymers such as polyethylene glycol (PEG), polyvinyl alcohol (PVA), polyacrylamide (PAM), poly (lactic-co-glycolic acid) (PLGA), and polycaprolactone (PCL). Synthetic hydrogels offer distinct advantages, including precise control over chemical composition, mechanical properties, degradation rates, swelling behavior, and bioactivity. This tunability allows synthetic hydrogels to be customized for highly specific drug delivery applications, ensuring consistent performance and reproducibility. For example, PEG-based hydrogels are widely utilized due to their inertness, flexibility in chemical modification, and controlled degradation properties, while PLGA hydrogels offer adjustable degradation rates suitable for sustained and targeted drug release [21,22,23].

Both chemical and physical hydrogels, as well as natural and synthetic hydrogels, possess unique yet complementary characteristics. The ability to tailor these properties through controlled synthesis and formulation techniques significantly expands their applicability in advanced drug delivery systems, particularly in addressing the clinical demands of effective and sustained analgesic therapies (Table 1).

### 2.2. Properties and Advantages of Hydrogels in Drug Delivery

#### 2.2.1. Biocompatibility and Biodegradability

A critical advantage of hydrogels in drug delivery is their exceptional biocompatibility and biodegradability. Hydrogels’ high-water content closely mimics natural tissue environments, thereby minimizing adverse immune responses and promoting favorable cellular interactions. Natural hydrogels, such as collagen, gelatin, alginate, and hyaluronic acid, are inherently biodegradable, undergoing enzymatic degradation into non-toxic byproducts easily cleared by physiological processes. This biodegradability facilitates their suitability for transient therapeutic applications, including wound healing, tissue engineering, and temporary scaffold support. Similarly, synthetic hydrogels can be precisely engineered with predictable degradation profiles through tailored polymer selection and crosslinking strategies, enabling controlled drug release and scaffold degradation to match specific clinical demands [18,24].

#### 2.2.2. Controlled and Sustained Drug Release

**Figure 1:**
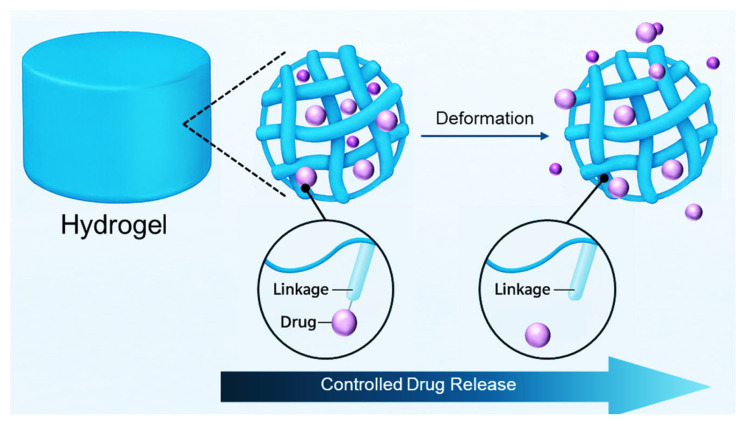
Schematic illustration of hydrogel-based controlled drug release.

Hydrogels uniquely excel in providing controlled and sustained drug release owing to their inherent structural properties. Their porous, hydrophilic polymeric networks facilitate the encapsulation and gradual diffusion of therapeutic agents, significantly prolonging drug bioavailability and stabilizing therapeutic concentrations at targeted sites. This prolonged, consistent release minimizes the need for repeated administrations, thereby enhancing patient adherence and reducing healthcare burdens. Furthermore, the structural properties and porosity of hydrogels can be finely adjusted through polymer selection, crosslinking density, and formulation methods, allowing precise modulation of release kinetics from hours to several months (Figure 1). Such controlled release is particularly advantageous in chronic pain management, postoperative analgesia, and prolonged therapeutic interventions [14,25].

#### 2.2.3. Responsive (Stimuli-Sensitive) Hydrogels

Responsive hydrogels, also termed stimuli-sensitive hydrogels, offer significant advantages through their capacity to undergo structural and physicochemical alterations in response to specific external or internal stimuli such as temperature, pH, enzymatic activity, ionic strength, light, and biomolecular signals. These dynamic responses facilitate precise, site-specific, and on-demand drug delivery. For instance, thermoresponsive hydrogels based on poly (N-isopropylacrylamide) (PNIPAAm) undergo reversible sol–gel transitions at body temperature, enabling minimally invasive injection methods and localized drug retention. Similarly, pH-responsive hydrogels incorporating acidic or basic groups release therapeutic agents selectively in response to pH changes characteristic of inflamed or tumorous tissues. Additionally, enzyme-responsive hydrogels containing specific substrate linkages can provide targeted drug release upon exposure to tissue-specific enzymes, offering further precision in therapeutic applications. Collectively, these responsive properties enhance drug efficacy, minimize systemic side effects, and significantly advance personalized medicine approaches in pain management and broader therapeutic contexts (Table 2) [26,27,28].

### 2.3. Current Clinical and Preclinical Applications

#### 2.3.1. Hydrogels in Local Anesthetic Delivery

Hydrogels represent a significant advancement in local anesthetic delivery, substantially enhancing the therapeutic efficacy, safety, and patient outcomes of anesthetic treatments. Preclinical studies have demonstrated the ability of hydrogel-based systems to sustain and precisely control the release of anesthetics such as bupivacaine, lidocaine, and ropivacaine. For example, alginate hydrogels loaded with bupivacaine have been extensively studied, revealing prolonged analgesia lasting several days post-administration, significantly reducing pain scores and opioid use in animal models undergoing surgical procedures. Chitosan-based hydrogels incorporating lidocaine have similarly demonstrated robust efficacy in preclinical models of chronic neuropathic pain, effectively extending the analgesic duration beyond traditional formulations and significantly decreasing systemic drug exposure and associated side effects [7,29].

#### 2.3.2. Clinical Perspective

Clinically, hydrogel-based local anesthetic systems have significantly enhanced patient care across various medical disciplines, including dentistry, orthopedics, dermatology, and wound management. Clinical trials involving lidocaine-loaded hydrogel formulations in dental surgeries have shown notable reductions in postoperative pain intensity, improved patient comfort, and decreased analgesic requirements. Similarly, orthopedic surgical procedures utilizing ropivacaine and bupivacaine encapsulated within hydrogels have demonstrated substantial postoperative analgesic benefits, reducing reliance on opioids and mitigating associated adverse events. Moreover, hydrogel-based anesthetic applications have proven highly effective in burn treatment and chronic wound care, not only offering effective pain control but also promoting enhanced wound healing, patient compliance, and overall therapeutic outcomes [7,30,31].

## 3. Liposomal Bupivacaine: A New Frontier in Pain Management

### 3.1. Pharmacology and Mechanism of Action

#### 3.1.1. Liposomal Encapsulation of Bupivacaine

Liposomal bupivacaine is an innovative formulation in which bupivacaine, a widely used amide-type local anesthetic, is encapsulated within lipid-based vesicles known as liposomes. These liposomes are composed of phospholipid bilayers that encapsulate aqueous compartments, providing structural integrity and protecting the active pharmaceutical ingredient. The encapsulation of bupivacaine into liposomes facilitates localized drug retention, thereby minimizing rapid systemic absorption and extending the duration of analgesic efficacy at the injection site. The lipid bilayers act as physical barriers, gradually releasing bupivacaine through mechanisms such as liposomal degradation, diffusion, and interaction with tissue fluids, significantly prolonging its therapeutic effects compared to conventional formulations [32,33,34].

#### 3.1.2. Extended Release and Pharmacokinetic Advantages

The pharmacokinetic advantages of liposomal bupivacaine are primarily attributed to its controlled and sustained-release properties. Unlike traditional bupivacaine solutions, which exhibit rapid systemic absorption and relatively short analgesic duration, liposomal bupivacaine provides prolonged drug release at therapeutic concentrations over a significantly extended period, often lasting up to 72 h post-administration. This sustained-release effect is achieved through gradual destabilization of the liposomal membranes, allowing slow, continuous diffusion of the encapsulated drug into surrounding tissues. Consequently, liposomal bupivacaine maintains steady-state therapeutic levels at the site of administration, thereby reducing fluctuations in plasma drug concentrations and minimizing systemic exposure. These pharmacokinetic characteristics effectively enhance analgesic efficacy, improve patient comfort, decrease the need for frequent dosing or supplemental analgesics, and substantially reduce the potential for systemic toxicity, making it highly beneficial in clinical pain management [35,36].

### 3.2. Clinical Applications and Effectiveness

#### 3.2.1. Efficacy in Postoperative Pain Management

Liposomal bupivacaine has demonstrated significant clinical efficacy in postoperative pain management across a variety of surgical disciplines. Clinical trials and studies have consistently reported improved postoperative analgesia, characterized by reduced pain scores, lower opioid consumption, and enhanced patient satisfaction. Specifically, in orthopedic procedures such as total knee arthroplasty, total hip replacement, and shoulder surgeries, liposomal bupivacaine has effectively provided prolonged analgesia, contributing to earlier mobilization, faster recovery times, and shorter hospital stays. In abdominal surgeries, including hernia repairs and colorectal procedures, the use of liposomal bupivacaine has significantly decreased the intensity of postoperative pain and minimized reliance on opioid medications, thereby reducing opioid-related adverse effects [37,38,39].

#### 3.2.2. Comparative Studies with Conventional Analgesics

Numerous comparative studies have highlighted the advantages of liposomal bupivacaine over conventional analgesics, including traditional bupivacaine formulations and systemic opioids. Clinical research comparing liposomal bupivacaine to conventional bupivacaine solutions has shown superior analgesic profiles, notably extending the duration of effective pain relief and enhancing patient comfort during the postoperative period. Additionally, studies evaluating liposomal bupivacaine against standard opioid-based pain management regimens have demonstrated substantial reductions in opioid consumption, mitigating risks associated with opioid use such as respiratory depression, nausea, constipation, and dependency (Table 3). Overall, evidence supports liposomal bupivacaine as a safer, more effective alternative to conventional analgesic therapies, improving patient outcomes and optimizing postoperative recovery [37,40].

### 3.3. Safety Profile and Limitations

#### 3.3.1. Adverse Events and Safety Considerations

The safety profile of liposomal bupivacaine is generally favorable, with most adverse events being mild to moderate in nature. Commonly reported adverse reactions include localized inflammation, swelling, erythema, and transient numbness at the injection site. Although rare, more severe reactions such as allergic responses, systemic toxicity, or neurological complications can occur, necessitating careful administration and patient monitoring. Additionally, despite the reduced systemic absorption provided by liposomal encapsulation, vigilance is required to monitor for potential local anesthetic systemic toxicity (LAST), particularly when higher doses or multiple injections are used [41,42].

#### 3.3.2. Economic Factors and Clinical Adoption Barriers

Despite its clinical advantages, widespread adoption of liposomal bupivacaine has been limited by economic considerations, primarily due to its relatively high upfront costs compared to traditional local anesthetics. The initial expense of liposomal formulations can pose significant budgetary constraints for healthcare facilities, particularly in resource-limited settings. Furthermore, insurance reimbursement variability and cost-effectiveness concerns often present additional barriers. Clinical adoption may also be hindered by limited awareness or familiarity among clinicians regarding the unique administration techniques required for optimal effectiveness. Overcoming these economic and educational barriers will be essential to fully realize the potential benefits of liposomal bupivacaine in routine clinical practice [43,44,45].

## 4. Combination of Hydrogels and Liposomal Bupivacaine

### 4.1. Rationale and Potential Synergistic Effects

#### 4.1.1. Enhanced Sustained-Release Capabilities

Combining hydrogels with liposomal bupivacaine leverages the complementary sustained-release characteristics of both systems, potentially offering superior and prolonged analgesic effects. Hydrogels provide a biocompatible, structurally supportive matrix capable of stabilizing liposomal vesicles, thereby protecting the encapsulated bupivacaine and ensuring controlled, gradual drug release. This integration can further optimize drug release kinetics by simultaneously employing liposomal membrane diffusion and hydrogel-mediated diffusion, significantly extending the duration of effective analgesia [9,46].

#### 4.1.2. Improved Local Analgesic Duration and Potency

**Figure 2:**
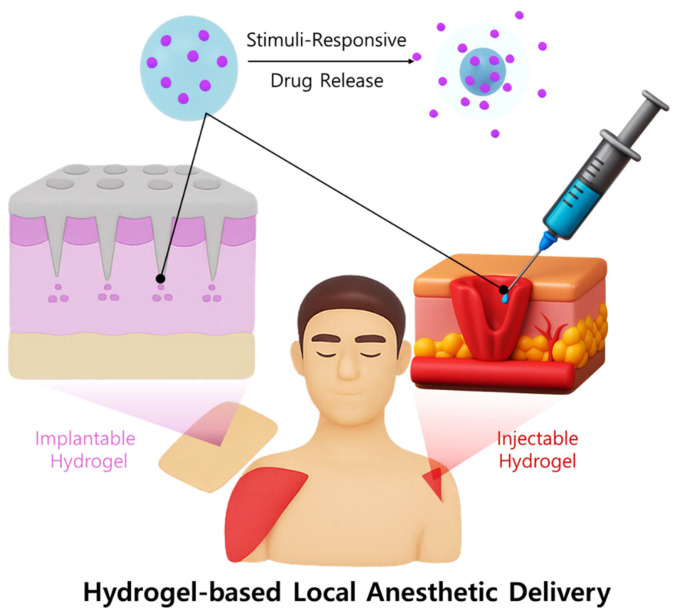
Schematic illustration of a hydrogel-based local anesthetic delivery system.

The synergistic combination of hydrogels and liposomal bupivacaine has the potential to markedly improve local anesthetic potency and prolong analgesic duration. Hydrogels maintain a localized drug reservoir, promoting consistent therapeutic concentrations at the targeted site, while liposomes enhance the encapsulation and slow release of bupivacaine (Figure 2). Consequently, this combined approach may reduce overall dosage requirements, minimize systemic exposure, enhance patient comfort, and significantly decrease the frequency of analgesic administration, thereby improving patient outcomes and safety in pain management [46,47,48,49].

### 4.2. Review of Existing Research

#### 4.2.1. Experimental Studies Combining Hydrogels and Liposomes

Recent experimental studies have extensively explored the integration of hydrogels with liposomal delivery systems, aiming to leverage the strengths of both technologies to create advanced drug delivery platforms. Various hydrogel matrices, including alginate, chitosan, gelatin, and thermosensitive PNIPAAm-based formulations, have been most frequently investigated owing to their biocompatibility, structural tunability, and controllable gelation behavior. In particular, alginate-liposome composites have exhibited sustained in vivo release of bupivacaine for up to 72 h, while chitosan hydrogels improved local drug retention and minimized systemic absorption. Similarly, gelatin-based and PNIPAAm hydrogels provided thermoresponsive sol–gel transitions that enable minimally invasive injection and in situ gelation at physiological temperatures. This hybridization of hydrogels and liposomes primarily seeks to enhance the controlled, localized release and bioavailability of therapeutic agents, offering significant improvements over traditional drug delivery approaches. For instance, liposome-loaded hydrogels have been investigated as carriers for various pharmaceuticals, including analgesics, antibiotics, and chemotherapeutic agents. These composite systems typically involve embedding liposomes within hydrogel matrices, which provides structural stability and controlled release profiles that significantly extend drug retention at the target sites [50,51].

Several studies have demonstrated the potential efficacy of these combined systems. Thermosensitive hydrogels incorporating thermoresponsive liposomes have been designed to undergo sol–gel transitions upon administration, allowing minimally invasive application with subsequent in situ gelation at physiological temperatures. This approach has shown promising results in preclinical models by achieving sustained and controlled drug release kinetics, effectively maintaining therapeutic concentrations at the site of administration over prolonged periods. Additionally, alginate- or chitosan-based hydrogels incorporating liposomal formulations of analgesics, such as bupivacaine, have been tested in animal models, showing marked extensions in analgesic duration and potency compared to conventional delivery systems, thus providing superior pain management outcomes [52,53,54].

#### 4.2.2. Outcomes, Efficacy, and Challenges

Before discussing the detailed outcomes, it is important to outline the principal limitations repeatedly reported in the literature. The most common challenges include (i) difficulty in achieving uniform distribution and stability of liposomes within hydrogel matrices, (ii) premature drug leakage during storage or early degradation, (iii) complexity of large-scale fabrication and reproducibility, and (iv) limited clinical translation due to regulatory hurdles. A brief overview of these issues helps contextualize the subsequent discussion of experimental outcomes and emphasizes the need for optimization in hybrid hydrogel liposome systems. The outcomes of experimental studies combining hydrogels with liposomes have consistently indicated substantial advantages in drug delivery efficiency. These hybrid systems facilitate sustained, localized drug release, minimizing systemic exposure and thereby significantly reducing adverse effects associated with traditional delivery methods (Table 4). For example, composite hydrogel-liposome systems for postoperative analgesia have demonstrated prolonged and stable analgesic effects in animal studies, highlighting their potential to reduce the reliance on systemic analgesics, including opioids [29,55,56].

Despite their promising therapeutic efficacy, several challenges persist, hindering the translation of these experimental platforms into clinical practice. One critical issue is the complexity involved in the fabrication of these hybrid systems. Ensuring uniform distribution and stability of liposomes within hydrogel matrices is technically demanding, and scaling up these processes for clinical-grade production is challenging. Additionally, maintaining long-term stability of liposomal encapsulation within hydrogels poses considerable technical hurdles, as factors like hydrogel degradation and premature drug leakage can adversely affect drug release profiles and therapeutic effectiveness. Moreover, regulatory considerations pose another significant barrier, as the multifaceted nature of these combined systems requires comprehensive safety evaluations and standardized manufacturing protocols to meet stringent regulatory requirements [55,57,58].

### 4.3. Potential Clinical Applications

#### 4.3.1. Surgical and Postoperative Pain Control

The combination of hydrogels and liposomal bupivacaine presents significant clinical potential, particularly in surgical and postoperative pain management. By integrating the sustained-release properties of hydrogels with the encapsulation capabilities of liposomes, clinicians can achieve prolonged analgesia with enhanced patient comfort and reduced systemic side effects. In orthopedic surgeries such as joint replacements, spinal procedures, and arthroscopic interventions, these composite systems could provide extended localized analgesia, reducing opioid consumption and facilitating earlier postoperative mobility. Similarly, in general surgical procedures, including hernia repair, colorectal surgery, and abdominal operations, the prolonged analgesic effects provided by hydrogel-liposome combinations may significantly improve postoperative pain management, reduce hospital stays, and enhance patient recovery experiences. Additionally, in plastic and reconstructive surgery, these innovative drug delivery systems could substantially mitigate pain and inflammation, promoting optimal healing conditions and patient satisfaction [48,59,60].

#### 4.3.2. Chronic Pain Management

Beyond acute and postoperative contexts, the synergistic use of hydrogels and liposomal bupivacaine offers considerable promise in managing chronic pain conditions. Chronic pain disorders, such as neuropathic pain, arthritis, or chronic inflammatory conditions, often present challenges due to their persistent nature and the limitations of current analgesic regimens. Hydrogel liposomal formulations could address these challenges by providing prolonged, targeted analgesia directly to affected sites, minimizing systemic exposure and associated adverse effects. For example, in chronic joint diseases like osteoarthritis, localized administration of hydrogel-encapsulated liposomal bupivacaine could deliver sustained relief, reducing the frequency of invasive interventions such as repeated intra-articular injections or systemic medications. Furthermore, in neuropathic pain conditions, targeted delivery systems could facilitate consistent and controlled analgesia, significantly enhancing patient quality of life and reducing the dependence on long-term opioid therapy. Despite the potential advantages, ongoing research is essential to optimize formulation characteristics, ensure safety and tolerability, and establish comprehensive guidelines for clinical implementation [7,61,62].

## 5. Technological Innovations and Recent Advances

### 5.1. Stimuli-Responsive Hydrogel Systems for Controlled Analgesia

**Figure 3:**
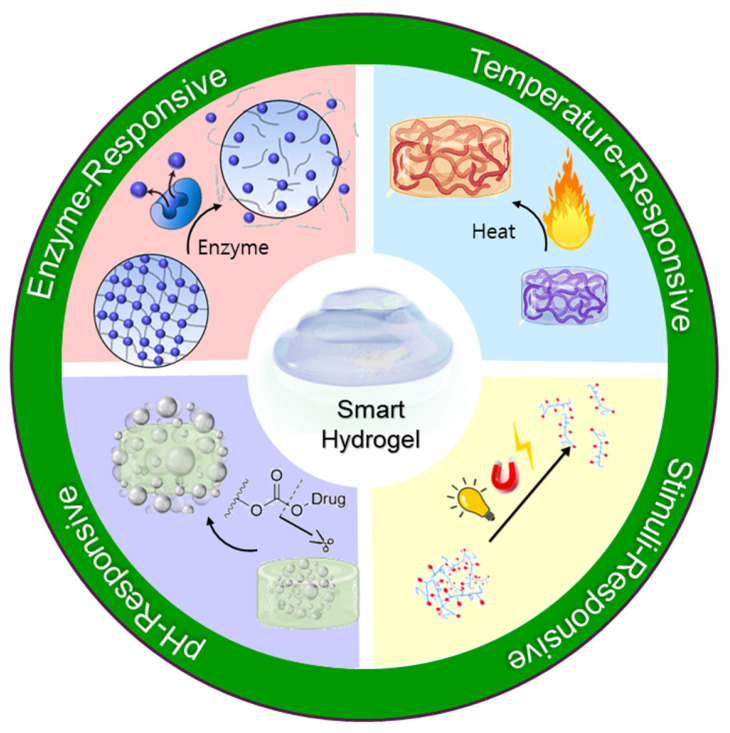
Schematic illustration of smart hydrogels and their representative stimuli-responsiveness.

Recent advancements in drug delivery technologies have highlighted the development of stimuli-responsive (“smart”) hydrogel systems, which significantly improve the precision and efficiency of analgesic drug release (Figure 3). These sophisticated hydrogels possess the unique capability to alter their physical or chemical structure in response to specific biological or environmental stimuli, enabling self-regulated, targeted drug delivery. Three major types temperature-responsive, pH-responsive, and enzyme-responsive hydrogels have been extensively investigated for their applicability in controlled analgesia [63,64,65].

#### 5.1.1. Temperature-Responsive Hydrogels

Temperature-sensitive hydrogels are designed to undergo reversible sol–gel phase transitions in response to changes in environmental temperature. These materials typically exist as injectable liquids at ambient temperatures, facilitating ease of administration, and rapidly transition into semi-solid gels at body temperature upon injection into the target tissue. This thermally induced gelation creates a depot capable of providing prolonged local analgesia through sustained release of encapsulated drugs, such as bupivacaine or lidocaine. Clinical and preclinical studies have reported extended analgesic durations and improved therapeutic outcomes using these temperature-responsive systems, particularly for postoperative pain management and regional anesthesia [9,48,66].

#### 5.1.2. pH-Responsive Hydrogels

Hydrogels sensitive to pH variations exploit the characteristic acidic microenvironment frequently observed in inflamed or injured tissues. When these hydrogels encounter areas of lowered pH, typical of inflammation sites, they respond by undergoing structural swelling or degradation, facilitating the controlled release of analgesic agents precisely at the locus of pain or inflammation. This selective responsiveness significantly enhances therapeutic efficacy and reduces unwanted systemic effects. For instance, recent preclinical models employing pH-sensitive hydrogels loaded with anti-inflammatory analgesics demonstrated site-specific drug delivery, reduced inflammation, and prolonged pain relief compared to conventional drug administration methods [67,68].

#### 5.1.3. Enzyme-Responsive Hydrogels

Enzyme-responsive hydrogels provide another innovative strategy for localized drug delivery by capitalizing on the heightened enzymatic activity in pathological states such as tissue injury or chronic inflammation. These hydrogels contain enzyme-specific peptide linkages or biodegradable polymer segments that degrade selectively in the presence of certain enzymes, including matrix metalloproteinases (MMPs), hyaluronidases, or proteases. When exposed to elevated enzymatic activity characteristic of inflamed or injured tissues, the hydrogels degrade in a controlled manner, releasing analgesics precisely at the targeted site. Recent experimental studies have demonstrated the potential of enzyme-responsive hydrogels in providing long-lasting pain control with minimal systemic exposure, highlighting their utility in both acute postoperative and chronic inflammatory pain scenarios [69,70].

#### 5.1.4. Self-Regulated Analgesic Release

The overarching advantage of stimuli-responsive hydrogel systems is their inherent capacity for self-regulated drug delivery, aligning analgesic release with physiological demands. By autonomously adjusting drug release rates based on dynamic physiological signals such as temperature shifts due to inflammation, pH fluctuations in damaged tissues, or enzymatic activity linked to pathological processes these hydrogel systems provide sophisticated, “smart” analgesic therapy. This responsive drug delivery not only optimizes therapeutic effectiveness but also significantly reduces the risk of side effects associated with systemic analgesics, ultimately improving patient safety, compliance, and quality of life [71,72,73].

### 5.2. Nanotechnology Integration in Hydrogels

#### 5.2.1. Nanoparticle-Enhanced Hydrogels

Recent innovations have increasingly focused on integrating nanotechnology into hydrogel-based drug delivery systems to overcome the limitations of traditional formulations. Nanoparticle-enhanced hydrogels utilize the unique characteristics of nanoparticles, such as their small size, high surface-to-volume ratio, and tunable surface chemistry, to significantly improve drug loading, controlled release, and therapeutic precision. Various nanoparticle types, including polymeric, metallic (e.g., gold or silver), silica-based, and lipid nanocarriers, have been incorporated into hydrogel matrices. This integration enables hydrogels to act as effective scaffolds that stabilize nanoparticles, protect encapsulated drugs from degradation, and ensure prolonged, controlled release over time [74,75].

#### 5.2.2. Improved Delivery Precision and Bioavailability

The combination of nanoparticles and hydrogels markedly enhances both the accuracy and bioavailability of local drug delivery. Nanoparticles embedded within hydrogels increase drug loading efficiency and allow adjustable release rates through controlled degradation or diffusion. Additionally, the modifiable surface chemistry of nanoparticles permits functionalization with targeting ligands or imaging agents, enabling precise delivery to specific tissues or cell populations. For example, nanoparticle–hydrogel composites loaded with analgesics exhibit deeper tissue penetration and longer residence times in pain-related regions, leading to superior therapeutic outcomes and reduced systemic exposure [76,77].

Furthermore, nanoparticle-enhanced hydrogels improve the solubility and stability of hydrophobic or poorly soluble analgesics, which are otherwise difficult to deliver effectively. By dispersing drug-loaded nanoparticles within hydrogel matrices, the overall drug solubility, local concentration, and therapeutic efficacy can be substantially enhanced. Preclinical studies consistently report prolonged analgesic duration, reduced dosage requirements, and fewer adverse effects compared with conventional formulations [7,29,61].

### 5.3. Printing and Personalized Medicine

#### 5.3.1. Customizable Hydrogel Delivery Systems

Three-dimensional (3D) printing technology has emerged as a transformative approach within pharmaceutical science, particularly for developing personalized hydrogel-based drug delivery systems. Utilizing advanced 3D printing techniques such as extrusion-based bioprinting, stereolithography, and inkjet printing, hydrogels can be precisely fabricated with patient-specific geometries, drug dosages, and release profiles. By customizing hydrogel structures at the microscale, clinicians and researchers can optimize therapeutic efficacy, tailor drug release kinetics, and minimize systemic adverse effects. Additionally, 3D-printed hydrogel matrices can incorporate multiple therapeutic agents simultaneously, enabling complex multi-drug therapies designed specifically to meet individual patient needs [78,79].

#### 5.3.2. Patient-Specific Analgesic Protocols

The integration of 3D printing technology with hydrogel-based delivery systems significantly advances the realization of personalized medicine, especially in analgesic therapies. Patient-specific analgesic protocols can now be effectively implemented by customizing hydrogels based on patient-specific parameters such as age, weight, severity and nature of pain, and anatomical differences. For instance, personalized hydrogel implants or patches can be precisely designed using patient imaging data (e.g., MRI or CT scans), enabling targeted drug delivery directly to affected tissues. This method enhances therapeutic precision, reduces the required drug dosages, and mitigates the risk of systemic toxicity, dramatically improving patient outcomes and satisfaction [7,80,81].

Recent preclinical studies have demonstrated that personalized 3D-printed hydrogel systems effectively maintain sustained analgesic concentrations in targeted tissues, outperforming conventional, non-customized treatments. These approaches have particular potential in managing complex chronic pain conditions, postoperative pain, and pain associated with degenerative diseases, where conventional therapies often fail to provide adequate relief [7,80,81].

**Figure 4:**
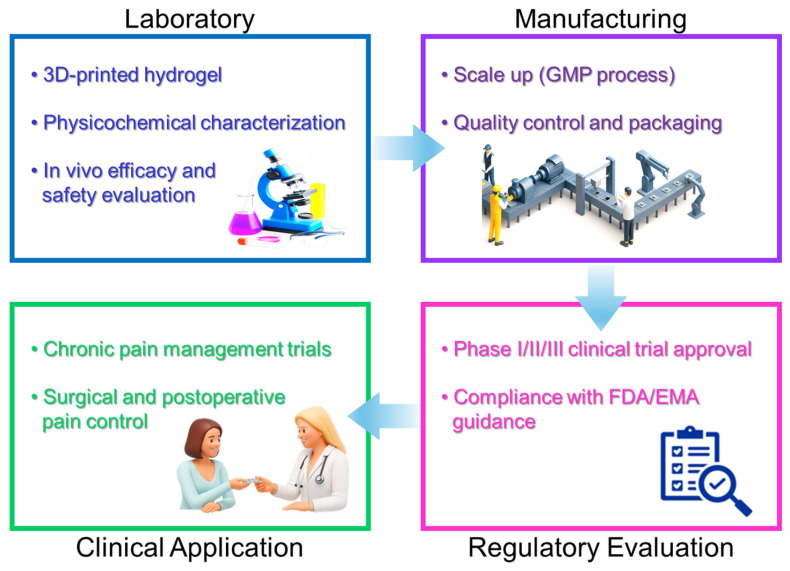
Clinical translation pathway of hydrogel–liposomal analgesic delivery systems, illustrating the progression from laboratory research to preclinical validation, large-scale manufacturing, regulatory evaluation, and clinical application.

However, the widespread clinical adoption of personalized 3D-printed hydrogel drug delivery systems faces several challenges, including regulatory hurdles, cost-effectiveness concerns, and standardization of production processes. Further advancements in biocompatible materials, reproducibility, and regulatory guidelines are crucial for the successful translation of personalized hydrogel analgesic therapies into routine clinical practice [82,83,84]. To better visualize this translational process, the overall progression from laboratory research to clinical implementation is summarized in Figure 4.

## 6. Challenges and Future Perspectives

### 6.1. Current Technological and Clinical Limitations

Despite the promising potential of advanced hydrogel systems and liposomal formulations in analgesic drug delivery, several technological and clinical limitations currently impede their widespread clinical adoption. Addressing these challenges is essential for ensuring safety, efficacy, and practical usability in real-world healthcare settings [7,55,85].

#### 6.1.1. Stability and Shelf-Life

One significant barrier involves ensuring the long-term stability and shelf-life of both hydrogel and liposome-based delivery systems. Hydrogels, particularly those that are stimuli-responsive or incorporate bioactive substances, can undergo structural or chemical degradation during storage, potentially compromising drug efficacy and safety. Similarly, liposomal formulations may suffer from aggregation, leakage of encapsulated drugs, or oxidation of lipids over extended storage periods. These stability concerns necessitate careful optimization of formulation parameters, storage conditions (such as temperature control and protection from environmental factors like humidity or oxygen), and the development of robust stabilization strategies to ensure consistency and reliability throughout the product lifecycle [86,87,88,89].

#### 6.1.2. Regulatory and Economic Challenges

Another critical obstacle to clinical translation involves regulatory approval processes and economic considerations. Due to the inherent complexity and novelty of advanced hydrogel liposomal drug delivery systems, regulatory agencies such as the FDA and EMA require extensive safety, efficacy, and quality-control data before granting market approval. These requirements include rigorous characterization studies, validation of manufacturing processes, and comprehensive clinical trials, which can be both time-consuming and costly [90,91].

Economically, the high initial costs associated with developing and producing novel hydrogel liposomal delivery platforms present additional barriers. The complexity of manufacturing processes often demands advanced equipment, specialized expertise, and stringent quality control, significantly increasing production costs. Furthermore, uncertainties regarding reimbursement policies and economic viability in healthcare systems may hinder widespread clinical adoption, particularly in regions with limited healthcare resources or budgetary constraints [92,93].

Addressing these technological, regulatory, and economic challenges requires continued interdisciplinary collaboration among scientists, clinicians, regulatory bodies, and industry stakeholders to streamline development processes, optimize cost-effectiveness, and establish clear regulatory guidelines (Table 5). Successfully overcoming these limitations will facilitate the broader clinical integration of these innovative drug delivery technologies, ultimately enhancing patient care and analgesic outcomes [94,95,96].

#### 6.1.3. Current Clinical Use and Ongoing Trials

Several hydrogel and liposomal local anesthetic formulations have already reached clinical or preclinical evaluation stages. Liposomal bupivacaine (Exparel^®^; Pacira BioSciences, Inc., Parsippany, NJ, USA) is currently approved by the U.S. FDA for postsurgical pain management in soft tissue and orthopedic procedures, with multiple ongoing studies investigating its efficacy in spine, colorectal, and reconstructive surgeries. Hydrogel-based bupivacaine delivery systems, including thermoresponsive and alginate matrices, are under investigation in phase I/II clinical trials for prolonged postoperative analgesia. Moreover, combined hydrogel-liposome systems are actively being evaluated in preclinical animal models, showing consistent analgesic effects with reduced systemic toxicity. These emerging data indicate the increasing translational readiness of these technologies, although further validation in large-scale human trials remains necessary.

### 6.2. Future Directions and Research Needs

The rapid advancement of hydrogel-based and liposomal analgesic delivery systems has opened numerous opportunities for innovation and improved patient outcomes. However, several key areas require further research and development to maximize their clinical potential [7,55,97].

#### 6.2.1. Improved Formulation Methods

Future research efforts should focus on developing and refining formulation methods to enhance the stability, reproducibility, and clinical efficacy of hydrogel-liposome hybrid systems. Advanced techniques such as microfluidics, electrospinning, and nanoencapsulation can offer precise control over hydrogel structure, porosity, and mechanical properties, significantly improving drug loading efficiency and release kinetics. Moreover, exploring novel biomaterials, particularly biodegradable, bioresponsive, and biocompatible polymers, could further optimize therapeutic performance while minimizing potential side effects. Additionally, innovative stabilization techniques, such as lyophilization or incorporation of stabilizing excipients, should be investigated to extend shelf-life and maintain product quality throughout storage and handling [55,98].

#### 6.2.2. Expansion of Clinical Trials and Patient Populations

A critical step toward widespread clinical adoption involves the expansion and diversification of clinical trials. Future clinical research should encompass larger, multicenter studies with diverse patient populations to thoroughly evaluate safety, efficacy, and clinical utility across various pain conditions and demographic groups. Special attention should be directed towards evaluating the long-term safety, cost-effectiveness, and patient-reported outcomes of these novel delivery platforms. Clinical trials designed to compare hydrogel liposomal systems directly against conventional analgesic therapies, across multiple clinical settings, would generate robust evidence supporting their integration into standard care practices. Furthermore, tailoring trials to investigate specific patient populations such as elderly individuals, pediatric patients, or patients with chronic comorbidities will enhance our understanding of how personalized and precise these systems can be, facilitating more targeted and individualized pain management strategies [99,100,101].

## 7. Conclusions

The integration of hydrogels with liposomal bupivacaine represents a significant advancement in pain management, offering innovative solutions to persistent challenges associated with conventional analgesic approaches. Combining these two advanced drug delivery technologies harnesses their complementary properties hydrogels providing sustained and controlled drug release, and liposomes enhancing drug stability, bioavailability, and duration of analgesia. This synergistic approach has demonstrated promising outcomes in preclinical and clinical studies, significantly improving therapeutic effectiveness, reducing systemic toxicity, minimizing opioid dependency, and ultimately enhancing patient comfort and recovery.

Despite these promising developments, challenges remain regarding formulation stability, large-scale manufacturing, regulatory approval, and economic feasibility. Addressing these barriers through ongoing interdisciplinary research and collaboration among scientists, clinicians, regulatory bodies, and industry stakeholders will be crucial to facilitate clinical translation.

Looking forward, advancements in stimuli-responsive hydrogels, nanoparticle-enhanced formulations, 3D printing, and personalized medicine hold substantial promise for further refining these combination systems. Expanded clinical trials involving diverse patient populations and robust comparative studies will be essential to validate efficacy, safety, and cost-effectiveness. Ultimately, continued innovation in hydrogel liposomal drug delivery systems holds the potential to significantly transform pain management practices, improve patient quality of life, and reduce healthcare burdens associated with inadequate pain control.

## Figures and Tables

**Table 1:** Classification of hydrogels based on crosslinking mechanism and polymer.

Category	Subtype	Key Features	Advantages	Limitations	Polymers
Crosslinking	Chemical hydrogels	Covalent bonds, permanent networks	High mechanical strength, long-term stability	Less reversible, potential toxicity of crosslinkers	PEG-diacrylate, PVA-crosslinked
	Physical hydrogels	Non-covalent	Reversible, stimuli-responsive	Weaker stability, shorter duration	PNIPAAm, PAA, chitosan-based
Origin	Natural hydrogel	Derived from biopolymers	Biocompatible, biodegradable, bioactive	Batch variability, weaker mechanics	Alginate, gelatin, collagen, HA
	Synthetic hydrogels	Engineered polymeric materials	Tunable properties, reproducible	Limited bioactivity	PEG, PLGA, PCL, PAM

**Table 2:** Key properties of hydrogels and corresponding advantages in drug delivery.

Property	Description	Clinical Advantages
Biocompatibility	High water content mimics the native tissue environment	Minimizes immune reactions, enhances patient safety
Biodegradability	Enzymatic or hydrolytic degradation into non-toxic byproducts	Temporary scaffolding avoids secondary surgery
Controlled/Sustained Release	Porous structure enables gradual diffusion	Prolonged analgesia, reduced dosing frequency
Stimuli-responsiveness	Structural changes triggered by pH, temperature, enzymes	Targeted, on-demand analgesia, reduced systemic side effects

**Table 3:** Clinical comparison between liposomal bupivacaine and conventional analgesics.

Parameter	Conventional Bupivacaine	Liposomal Bupivacaine	Opioids
Duration of analgesia	4–8 h	Up to 72 h	Variable (short- to long-acting)
Opioid-sparing effect	Minimal	Significant	None
Safety profile	Systemic toxicity at high/repeated doses	Lower systemic absorption, fewer adverse events	High risk of respiratory depression, dependency
Administration	Requires repeated injections or catheters	Single-dose infiltration sufficient	Oral/IV, systemic
Cost considerations	Low	High upfront cost, potential long-term savings	Variable, but often costly due to complications

**Table 4:** Advantages and limitations of hydrogel–liposome hybrid systems.

Aspect	Advantages	Limitations
Drug release	Dual sustained-release kinetics	Complex formulation control
Clinical efficacy	Prolonged and potent analgesia, localized effect	Limited large-scale clinical evidence
Safety	Reduced systemic exposure, fewer side effects	Stability issues, risk of premature leakage
Practical use	Potential to reduce opioid reliance	Fabrication complexity, regulatory barriers

**Table 5:** Summary of technological, regulatory, and economic barriers.

Barrier	Key Issues	Impact
Stability/ Shelf-life	Hydrogel degradation, liposome leakage, lipid oxidation	Loss of drug potency, reduced reliability
Manufacturing	Scale-up complexity, uniform distribution of liposomes	Limited reproducibility, high production cost
Regulatory	Extensive safety/efficacy trials required	Delayed clinical translation
Economic	High upfront cost, uncertain reimbursement	Limits adoption in resource-constrained settings
Clinical adoption	Need for training, unfamiliar administration techniques	Slower acceptance among practitioners

## Data Availability

The datasets generated and/or analyzed during the current study are not publicly available because disclosing patients’ personal information is against the law but only de-identified datasets are available from the corresponding author on reasonable request.
